# HIRA contributes to zygote formation in mice and is implicated in human 1PN zygote phenotype

**DOI:** 10.1530/REP-20-0636

**Published:** 2021-04-08

**Authors:** Rowena Smith, Sue J Pickering, Anna Kopakaki, K J Thong, Richard A Anderson, Chih-Jen Lin

**Affiliations:** 1 MRC Centre for Reproductive Health, University of Edinburgh, Queen’s Medical Research Institute, Edinburgh Bioquarter, Edinburgh, UK; 2 Edinburgh Fertility and Reproductive Endocrine Centre, Simpson’s Centre for Reproductive Health, Edinburgh Royal Infirmary, Edinburgh, UK

## Abstract

Elucidating the mechanisms underpinning fertilisation is essential to optimising IVF procedures. One of the critical steps involves paternal chromatin reprogramming, in which compacted sperm chromatin packed by protamines is removed by oocyte factors and new histones, including histone H3.3, are incorporated. HIRA is the main H3.3 chaperone governing this protamine-to-histone exchange. Failure of this step results in abnormally fertilised zygotes containing only one pronucleus (1PN), in contrast to normal two-pronuclei (2PN) zygotes. 1PN zygotes are frequently observed in IVF treatments, but the genotype-phenotype correlation remains elusive. We investigated the maternal functions of two other molecules of the HIRA complex, *Cabin1* and *Ubn1*, in mouse. Loss-of-function *Cabin1* and *Ubn1* mouse models were developed: their zygotes displayed an abnormal 1PN zygote phenotype. We then studied human 1PN zygotes and found that the HIRA complex was absent in 1PN zygotes that lacked the male pronucleus. This shows that the role of the HIRA complex in male pronucleus formation potentially has coherence from mice to humans. Furthermore, rescue experiments in mouse showed that the abnormal 1PN phenotype derived from *Hira* mutants could be resolved by overexpression of HIRA. We have demonstrated that HIRA complex regulates male pronucleus formation in mice and is implicated in humans, that both CABIN1 and UBN1 components of the HIRA complex are equally essential for male pronucleus formation, and that rescue is feasible.

## Introduction

Fertilisation of an oocyte by a sperm to produce a zygote and then an embryo underlies mammalian reproduction. As a treatment for infertility, IVF is well established, but although fertilisation remains at its centre, our understanding of this process is limited, preventing the possibility of specific diagnoses and thus treatments that may be relevant to some couples, and which may improve success rates overall. To achieve a successful fertilisation event, consequential steps including oocyte activation, chromatin reprogramming of the sperm (protamine-to-histone exchange and histone reassembly), and formation of the pronuclei are essential. These all rely on the quality of the oocytes, and this is dependent on oocyte stored factors (Gosden 1996, Swain & Pool 2008). Therefore, it is imperative to elucidate the underlining regulatory mechanisms involved in these steps, in the search for potential new IVF treatments which can overcome defects found in poor quality oocytes. An intriguing clinical phenotype arising from abnormal fertilisation is the one-pronucleus (1PN) zygote. It is observed after overnight culture following both conventional IVF and ICSI and occurs in 3–17% of fertilised oocytes, though mostly in the range of 4–8% (Azevedo *et al.* 2014, Si *et al.* 2019). However, the aetiology is unknown.

The building blocks of chromatin are the core histones H2A, H2B, H3, and H4. In addition to these canonical histones, there are variants which share similar sequences with canonical histones and which carry out diverse functions. One example is histone variant H3.3, a version of H3 with wider regulatory roles, one of which, significantly, is that it allows H3.3 to bind to chromatin independently of the cell cycle (Maze *et al.* 2014). The incorporation of histones onto chromatins is tightly regulated by histone chaperones (Buschbeck & Hake 2017, Hammond *et al.* 2017). HIRA complex, which comprises HIRA, CABIN1, and UBN1, is one of the H3.3 chaperones (Rai *et al.* 2011).

The role of H3.3 incorporation during fertilisation and the underlying mechanism of mediation by histone chaperone HIRA, have been reported in model organisms such as drosophila (Loppin *et al.* 2005) and carp (Zhao *et al.* 2011). However, direct evidence in mouse had not been reported until a few years ago (Inoue & Zhang 2014, Lin *et al.* 2014, Tang *et al.* 2015). Applying a mouse genetic model, we reported that maternal *Hira* is essential for the completion of chromatin reprogramming of the sperm during fertilisation; mutant *Hira* oocytes formed abnormal 1PN zygotes that lacked a male pronucleus due to the failure of H3.3 incorporation (Lin *et al.* 2014). However, the maternal roles of the other molecules in the *Hira* complex, *Cabin1* and *Ubn1*, remain unknown in mammals.

In this study, we explored the maternal role of the remaining candidates within the HIRA complex, *Ubn1* and *Cabin1*, in the mouse. We have found that they are essential in the paternal chromatin reprogramming event. Loss-of-function mouse models for either *Ubn1* or *Cabin1* exhibited the same 1PN phenotypes as *Hira* mutants, and rescue experiments revealed that the abnormal 1PN phenotype of *Hira* mutants could be restored back to 2PN zygotes.

Our examination of abnormal human 1PN zygotes showed that failure of HIRA complex binding is possibly involved in the fertilisation cascade steps similar to the mouse.

## Materials and methods

### Animals

Mouse experiments were approved by the University of Edinburgh’s Animal Welfare and Ethical Review Board (AWERB) and carried out under the authority of a UK Home Office Project Licence. The mouse lines used in this study carried conditional floxed alleles for *Cabin1* and for *Hira* on C57BL/6 backgrounds (both were provided from Prof Peter Adams previously of the Beatson Institute, UK, now at Sanford Burnham Prebys, USA). Floxed *Cabin1* (Cabin*1* fl/fl) and floxed *Hira* (*Hira* fl/fl) mouse lines were crossed with a Zp3-Cre mouse line ((de Vries *et al.* 2000); provided from Prof Petra Hajkova, Imperial College, London) that expresses Cre recombinase in the female germline to generate heterozygous and homozygous mutant oocytes.

### Manipulation of oocytes and embryos

Female mice were primed by administration of 7.5 I.U. pregnant mare serum gonadotrophin (PMSG from Prospec Protein Specialists, USA) or 80 µL ultra-superovulation reagent (CARD HyperOva Cosmo Bio, Japan; (Hasegawa *et al.* 2016)) and 48 h later fully grown oocytes were isolated. For *in vitro* maturation (IVM), oocytes were cultured in M16 medium (Sigma) at 37°C, 6% CO_2_ and 5% O_2_ for 18 to 24 h.

For the zygote collection, 48 h after PMSG priming, 7.5 I.U. human chorionic gonadotrophin (hCG, Chorulon® from Intervet) was injected and the mice mated with C57BL/6 males. The following day zygotes were collected from the oviducts and cumulus cells were removed after treatment with 300 μg/mL hyaluronidase (Millipore).

The microinjection procedure was the same as previously described (Chen *et al.* 2013, Lin *et al.* 2014). The micromanipulation platform was equipped with a microinjector (FemtoJet 4i, Eppendorf), an inverted microscope (Leica, DMi8), and micromanipulators (Narishige). For *Ubn1* knockdown experiments, oocytes were microinjected with 2 mM of either control or *Ubn1* antisense morpholino oligos (Gene Tools). Injected oocytes were cultured in M16 for subsequential *in vitro* maturation and *in vitro* fertilisation experiments. For validation of knock-down by immunofluorescence, oocytes were cultured overnight in M16+IBMX to retain the GV stage.

For the HIRA rescue experiments, *Hira-GFP* (a gift from D Dutta) and *GFP* (a gift from M Anger, IAPG, CAS) were used as templates for *in vitro* synthesis of RNA using mMessage mMachine® (Ambio) followed by polyadenylation using a Poly(A) tailing kit (Applied Biosystems). Transcripts were purified by phenol:chloroform extraction and isopropanol precipitation and diluted to 160 ng/µL for microinjection.

*In vitro* fertilisation(IVF) was performed as previously described (Sztein *et al.* 2000) with minor modifications. Briefly, sperm were collected from the vasa deferentia and then capacitated for 1.5 h in human tubal fluid (HTF) medium (Millipore). Sperm cells were then incubated with intact cumulus-oocyte complexes (final sperm concentration ~1.4 × 10^5^/mL) or zona-free oocytes (for rescue experiments; final sperm concentration ~3.1 × 10^5^/mL) for 5 h. After removing excess sperm and cumulus lysate, presumptive fertilized eggs were transferred to equilibrated KSOM+AA medium (Millipore) for further culture and analysis. In order to facilitate the IVF efficiency and pronuclear formation of our C57BL/6 inbred strain, we adapted the culture conditions by using low oxygen concentrations, similar to Sztein *et al.* (2000). Specifically, we used a benchtop incubator for IVF and embryo culture with 6% CO_2_, 5% O_2_, and N_2_.

### Immunohistochemistry (IHC)

IHC was carried out according to standard protocols. Briefly, ovaries from 4 to 8-week-old mice were fixed in 4% paraformaldehyde overnight at 4°C then washed for 3 × 10 min in PBS followed by transfer to 70% ethanol before being processed into paraffin wax blocks. Sections of 5 µm were cut and mounted onto SuperfrostPlus slides (Thermo Fisher Scientific). After de-waxing and rehydration, slides were subject to antigen retrieval by pressure cooking at maximum pressure for 5 min in 0.01M sodium citrate buffer pH 6.

To reduce non-specific staining from endogenous peroxidases, sections were incubated in 3% hydrogen peroxide (Sigma) solution for 15 min.

Prior to incubation with the primary antibody, incubation with normal serum and BSA was carried out to reduce non-specific binding. The brown signal was revealed after incubation with ImmPRESSTM HRP Anti Rabbit IgG (Peroxidase) Polymer detection kit followed by ImmPACTTM DAB Peroxidase (HRP) substrate (both from Vector laboratories).

The sections were counterstained with haematoxylin and imaged on a Zeiss AxioImager Z1.

### Immunofluorescence (IF)

IF experiments were performed as previously reported (Lin *et al.* 2014). Most oocytes/embryos were directly fixed in 4% paraformaldehyde for 15 min. To remove unbound histones, some oocytes were incubated with pre-extraction buffer containing 0.5% Triton X-100 for 5 min, following Hajkova and Nashun protocol (Paull *et al.* 2013), before being fixed in 4% paraformaldehyde. Fixed samples were permeabilised by 0.5% Triton X-100 and then blocked by 5% BSA before incubation with primary antibodies. The antibodies used were HIRA (LSBio LS-C137477), UBN1 (a gift from Peter Adams), H3.3 (Abnova H00003021-M01), CABIN1 (Abcam ab3349), H3K9me2 (Abcam ab1220), H3K9me3 (Abcam ab8898). Secondary antibodies used were donkey anti rabbit IgG, 568 nm (Thermo Fisher A10042), and donkey anti mouse IgG, 488 nm (Thermo Fisher A21202).

After washing and counterstaining with DAPI (Life Technologies D3571), samples were mounted using Vectashield® (Vector laboratories).

Images of stained oocytes/embryos were acquired by a spinning disk confocal (CSU-W1, Yokogawa) on an upright microscope frame (BX-63, Olympus) using a 30 or 60× silicon oil immersion objective (UPLSAPO 60XS2, Olympus) (Percharde *et al.* 2018). IF signal intensity was quantified using Fiji software.

### Proximity ligation assay (PLA)

Proximity ligation assay was performed according to the instructions of the PLA Duolink® assay kit manual (Sigma). Briefly, WT zygotes (2- and 4-h post IVF) were fixed for 15 min in 4% paraformaldehyde followed by permeabilisation for 20 min in 0.5% Triton X-100 in PBS. The samples were then blocked in PLA Duolink® blocking solution before being incubated with primary antibodies: either mouse anti HIRA (LSBio LS-C137477) and rabbit anti UBN1(a gift from Peter Adams) or mouse anti HIRA and rabbit anti CABIN1 (Abcam ab3349). As a negative control the antibodies raised in rabbit were incubated with mouse anti hCG (Abcam ab9582) which should not be present in mouse tissues.

All wash steps were with wash buffer A. After washing, the samples were incubated with the PLA PLUS and MINUS probes, diluted as directed, for 60 min at 37°C. Following washing, the samples were incubated for 30 min at 37°C in ligation reaction mixture followed by further washes before being incubated for 100 min at 37°C in the reaction mixture for rolling circle amplification. The samples were mounted on SuperfrostPlus® slides (Thermo Fisher Scientific) using Duolink® mounting medium plus DAPI. Images were acquired by a spinning disk confocal (CSU-W1, Yokogawa) on an upright microscope frame (BX-63, Olympus) using a 30 or 60× silicon oil immersion objective (UPLSAPO 60XS2, Olympus). Quantification of interaction foci was performed using Fiji software.

### Human one-pronucleus zygotes

Approvals were obtained from an ethical committee (East of Scotland Research Ethics Service; REC 16/ES/0039) and the Human Fertilisation and Embryology Authority (HFEA), research licence (R0204), for the collection of human one-pronucleus (1PN) zygotes, in collaboration with the Edinburgh Fertility and Reproductive Endocrine Centre (EFREC). Patients were recruited before oocyte collection, and samples were collected from couples who gave informed consent, in writing.

Oocyte and sperm collection, fertilisation by IVF or ICSI, and embryo culture procedures, were performed following the routine operational protocols of EFREC and described in Sciorio * et al.* (Sciorio *et al.* 2018). Briefly, cumulus-oocyte-complexes (COC) were retrieved from follicular fluid, and then oocytes were isolated and washed. Oocytes were inseminated by IVF or ICSI as clinically indicated. Inseminated oocytes were cultured in G-IVF Plus medium (Vitrolife, Sweden) at 37°C and 6% CO_2_ in atmospheric air. Single pronucleus zygotes were identified approximately 18 h post IVF or ICSI.

Embryo disposal, transfer, and witness documents followed the regulations of the HFEA clinic licence (0201). 1PN zygotes were individually labelled and transported using a portable incubator in G-MOPS Plus medium (Vitrolife, Sweden) at 37°C from the clinic laboratory to the research laboratory. Zygotes were then fixed in 4% paraformaldehyde for 15 min at room temperature. IF procedure and confocal imaging were the same as described above.

Antibody against H3K9me3 was used as a marker of human female chromatin (van de Werken *et al.* 2014) and UBN1, HIRA, H3.3, and CABIN1 antibodies were also applied.

### Statistical analyses

For analyses of the proportion of pronuclear formation, the χ^2^ test was used. For analyses of IF intensity, two-tailed *t*-test with unequal variance was used. ANOVA was used for PLA quantification. All error bars indicate s.d..

## Results

### Maternal HIRA complex is involved in male chromatin deposition in mouse oocytes

**Figure 1:**
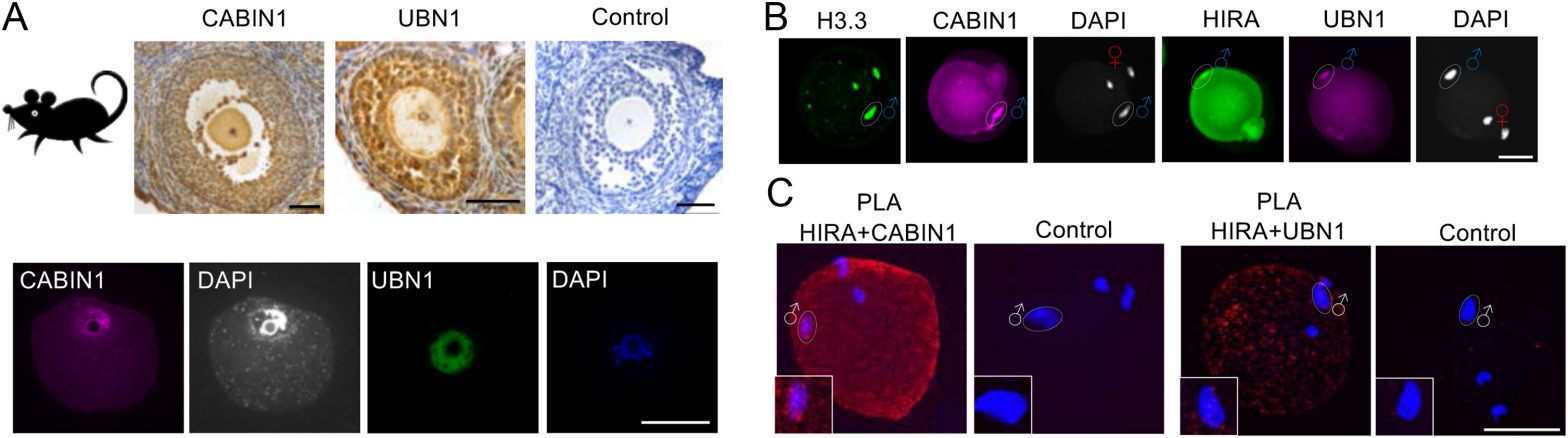
Maternal HIRA complex associates with male chromatin after fertilisation. (A) CABIN1 and UBN1 are maternal factors present in the mouse oocytes. Immunohistochemisty of murine ovarian sections (upper panel) and immunofluorescence of GV oocytes (lower panel) showed positive signals of CABIN1 and UBN1 in the GV nuclei. Scale bar = 50 μm. (B) CABIN1 and UBN1 bind to male chromatin after fertilisation. Immunofluorescence of H3.3 and CABIN1 (left panel) and HIRA and UBN1 (right panel) show positive signals in the decondensed male chromatin after fertilisation. Scale bar = 50 μm. (C) Proximity ligation assay (PLA) confirmed the interactions of CABIN1with HIRA (left panel) and UBN1with HIRA (right panel) in the male chromatin during sperm decondensation. Left panel: images of embryos stained with antibodies against HIRA and CABIN1. Negative control: antibody of hCG and CABIN1; Right panel: images of embryos stained with antibodies against HIRA and UBN1. Negative control: antibody of hCG and UBN1; Inset: enlarged (1.7-fold) images of male chromatins. Scale bar = 50 μm.

First, we examined whether the constituent molecules of the HIRA complex were present in WT mouse oocytes during oogenesis. Immunohistochemistry of mouse ovarian sections demonstrated that both CABIN1 and UBN1 were present in oocytes and were particularly enriched in germinal vesicle (GV) nuclei (Fig. 1A). Wholemount immunofluorescence (IF) of GV stage oocytes also confirmed this observation (Fig. 1A). Therefore, both CABIN1 and UBN1 are oocyte factors that accumulate in GV oocytes.

We postulated that both CABIN1 and UBN1 collaborate with HIRA and are involved in the chromatin reprogramming processes during fertilisation. We collected and fixed newly fertilised IVF embryos (2–4 h post fertilisation, PN 0 stage) in which female chromatin had completed the 2nd meiotic division and in doing so had segregated sister chromatids. Meanwhile, male chromatin had undergone DNA decondensation. IF showed that CABIN1 had deposited into chromatin and co-localised with H3.3. UBN1 had also deposited into male chromatin and showed a similar pattern to HIRA (Fig. 1B; (Lin *et al.* 2014)).

To provide further evidence of the interactions within HIRA complex during male chromatin decondensation, we performed proximity ligation assay (PLA), a sensitive assay for visualisation of protein–protein interactions. First, we examined the interaction of UBN1 with HIRA and CABIN1 with HIRA in the GV oocytes, respectively. We detected significantly more positive PLA signals in the nuclei of both UBN1 with HIRA and CABIN1 with HIRA groups than either UBN1 or CABIN1 alone or with hCG (a protein which is absent in the mouse) antibody (Supplementary Fig. 1, see section on supplementary materials given at the end of this article). Next, we examined the PN0 stage zygotes and detected positive signal foci for HIRA and CABIN1 and HIRA and UBN1 in the decondensed male chromatin, but not in the negative controls (Fig. 1C). We also observed foci for HIRA and CABIN1 as well as HIRA and UBN1 distributed throughout the zygotes. It suggests that CABIN1 and UBN1 potentially interact in the cytoplasm and contribute to oocyte developmental competence. As we previously reported, HIRA has a dual role for impacting on male pronuclear formation and zygotic genome activation (Lin *et al.* 2014) and (Smith *et al.* 2020). Thus, we demonstrated that both CABIN1 and UBN1 are maternal factors deposited in oocytes and associate with decondensed male chromatin after fertilisation and that they physically interact with HIRA. We hypothesised that the molecules within the HIRA complex could collaboratively play a role during paternal chromatin reprogramming.

### HIRA complex is essential for male pronucleus formation during fertilisation in mice

We then generated three new HIRA complex models, each one with oocyte loss-of-function in one component part of the HIRA complex (Supplementary Fig. 2A).

**Figure 2:**
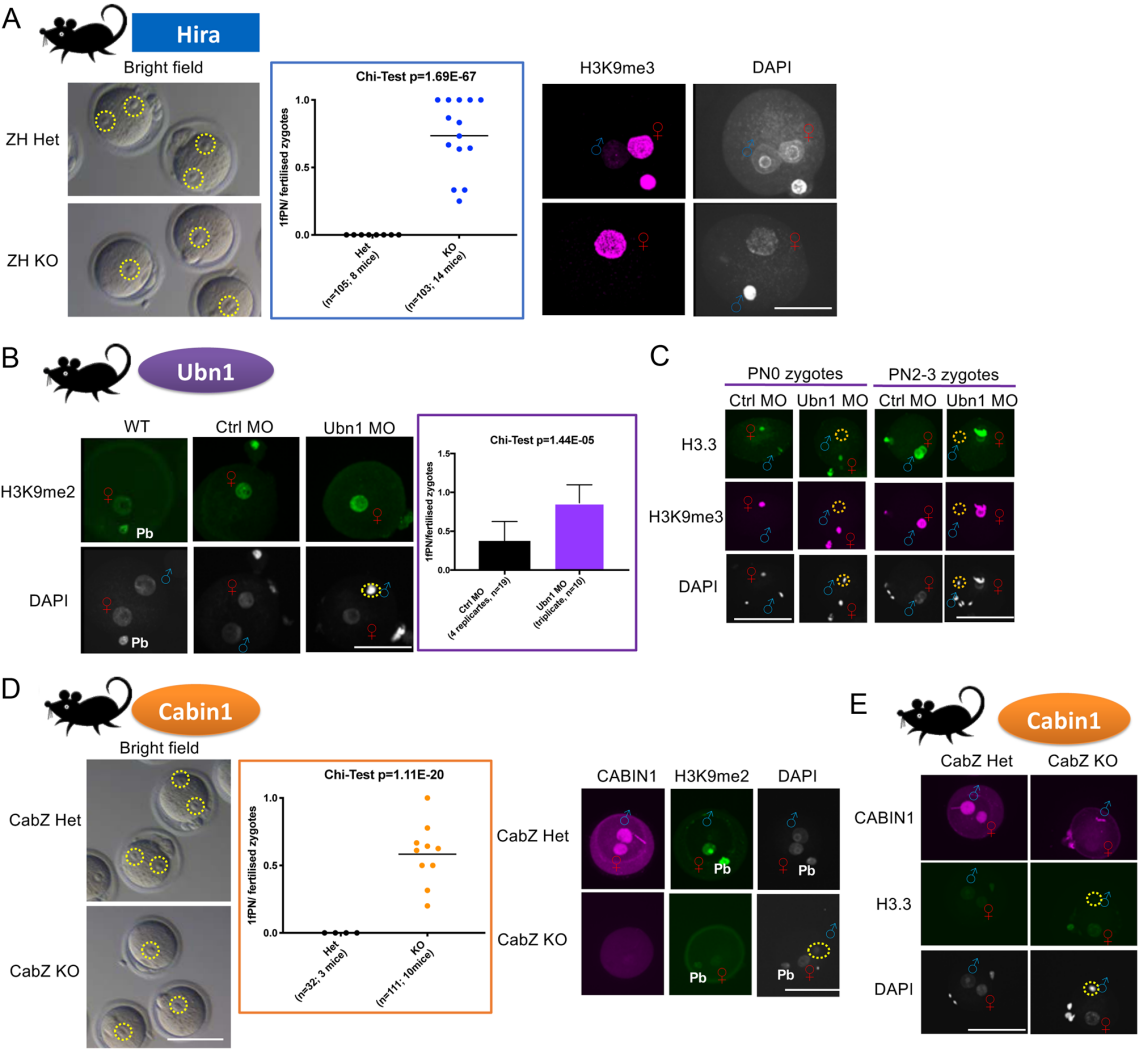
Loss-of-function maternal HIRA complex in mice gave rise to abnormal one-pronucleus (1PN) zygotes due to failure to form male pronuclei. (A) *Hira* mutant oocytes formed abnormal 1PN zygotes after fertilisation. Brightfield images showed abnormal 1PN zygotes of *Hira* mutants (ZH KO), in contrast to normal two-pronuclei (2PN) zygotes in the controls (ZH Het; left panel). Abnormal 1PN formation rate was increased in the *Hira* mutant zygotes (middle panel). Immunofluorescence showed that male pronucleus formation is impaired in *Hira* mutant zygote (right panel). Female chromatin was labelled with histone H3K9me3. Scale bar = 40 μm. (B) *Ubn1* knockdown oocytes formed 1PN zygotes after fertilisation. Male pronucleus formation is impaired in *Ubn1* morpholino (MO) knocked down zygotes. Histone H3K9me2 stained for female chromatin (left panel). Abnormal 1PN formation rate was increased in the Ubn1MO knockdown zygotes (right panel). Scale bar = 40 μm. (C) Histone H3.3 failed to incorporate into male chromatin in the *Ubn1* knockdown zygotes. Left panel: in *Ubn1* knockdown embryos immediately after fertilisation (pronuclear stage 0, PN0), H3.3 failed to incorporate into male chromatin which was undergoing the decondensation process. Right panel: H3.3 failed to incorporate into male chromatin in* Ubn1* knockdown zygotes at PN2-3 stage. Histone H3K9me2 stained for female chromatin. Scale bar = 80 μm. (D) *Cabin1* mutant oocytes formed abnormal 1PN zygotes after fertilisation. Brightfield images showed abnormal 1PN zygotes formed in the *Cabin1* mutants (CabZ KO), in contrast to normal 2PN zygotes in the controls (CabZ Het; left panel). Abnormal 1PN formation rate was increased in the *Cabin1* mutant zygotes (middle panel). Immunofluorescence showed that male pronucleus formation is impaired in the *Cabin1* mutants. Female chromatin was labelled with histone H3K9me2 (right panel). Scale bar = 80 μm. (E) Histone H3.3 failed to incorporate into male chromatin in the *Cabin1* mutant zygotes. Scale bar = 80 μm.

We generated a new *Hira* conditional knockout mouse line different from our previous report (Lin *et al.* 2014). We used a Zp3-Cre mouse line to inactivate the *Hira* flox/flox alleles and so delete exon 6–7. We validated the loss of *Hira* mRNA in the GV oocytes by qRT-PCR and the significant reduction of HIRA in both GV oocytes and zygotes confirmed by IF which were similar to our previous report (Lin *et al.* 2014; data not shown). In addition, we observed an infertile phenotype in the new *Hira* mutant mice which was consistent with that seen in our previous model (no pups were generated from four mutant females mated with proven stud males for over 4 months; compared with an average litter size of six from heterozygous control; data not shown). Zygotes retrieved from this new *Hira* mutant also displayed 1PN phenotypes due to the failure of male pronucleus formation (73.5%; Fig. 2A).

For *Ubn1*, we applied the morpholino antisense oligos knockdown approach to oocytes ((Lin *et al.* 2013, 2014); Supplementary Fig. 2B). After injection of morpholino oligos against *Ubn1* (Ubn1 MO), the level of UBN1 in oocytes was significantly reduced compared to control oligos (Ctrl MO) (Supplementary Fig. 2C; 49% reduction of UBN1 after knockdown). Interestingly, fertilised Ubn1 MO matured oocytes revealed a high proportion (87%) of abnormal 1PN zygotes (Fig. 2B). To examine whether H3.3 can incorporate into male chromatin in *Ubn1* KD zygotes, we performed IF on PN0 and PN2-3 stages zygotes, respectively. After fertilisation, H3.3 was incapable of incorporating into male chromatin during the sperm decondensation process (PN0 stage) in *Ubn1* KD zygotes. Later, it fails to incorporate into the male pronucleus (PN2-3 stage). In contrast, H3.3 incorporated into male chromatin in both PN0 and PN2-3 stage zygotes of controls (Ctrl MO) (Fig. 2C).

To investigate the maternal role of *Cabin1*, we again used the Zp3-Cre approach and crossed to *Cabin1*flox/flox mice to delete exon 6 (Supplementary Fig. 2A). *Cabin1* mutant mice (CabZ KO) also showed an infertile phenotype, and fertilised CabZ KO zygotes revealed the abnormal 1PN phenotype due to the failure of male pronucleus formation (Fig. 2D). IF on CABIN1 zygotes demonstrated that H3.3 was incapable of incorporating into male chromatin in CabZ KO oocytes. In contrast, H3.3 incorporated into pronuclei of *Cabin1* heterozygous controls (CabZ Het) (Fig. 2E).

Since UBN1 has been reported as providing the specificity of H3.3 binding (Ricketts *et al.* 2015) and CABIN1 functions as enhancing and stabilising the overall HIRA complex (Ray-Gallet *et al.* 2018), we also investigated whether the loss-of-function of *Ubn1* and *Cabin1* compromised the overall stability of the HIRA complex in the oocytes. We performed IF on *Ubn1* knockdown oocytes and assessed the relative level of HIRA and CABIN1, the two other molecules within the HIRA complex. We found that the overall nuclear staining signals of HIRA and CABIN1 were significantly decreased in Ubn1 MO oocytes compared to Ctrl MO oocytes (Supplementary Fig. 2D). This indicated that the loss-of-function of HIRA complex resulted in mis-localisation in the nucleus which suggests the decrease of binding stability. We also performed the Triton X-100 pre-extraction protocol to remove the unbound chromatin-associated signals in Ubn1 KD oocytes (Nashun *et al.* 2015), and noted that the level of H3.3 was comparably lowered in these oocytes (Supplementary Fig. 2D).

Similarly, we conducted IF on CabZ KO oocytes. Not only were overall nuclear staining signals of UBN1 and HIRA reduced in CabZ KO oocytes but so was the level of H3.3 (Supplementary Fig. 2E). We also performed the Triton X-100 pre-extraction in CabZ oocytes, and noted that the level of H3.3 was comparably lowered in CabZ KO oocytes (Supplementary Fig. 2E).

The above results suggest that each molecule of HIRA complex is essential for the stability of the complex in the oocyte and loss of any one of them affects male pronucleus formation at fertilisation.

### HIRA complex failed to associate with male chromatin of human abnormal 1PN zygotes

Based on the promising data collected from mice, we investigated whether the HIRA complex is involved in male pronucleus formation in humans.

**Figure 3:**
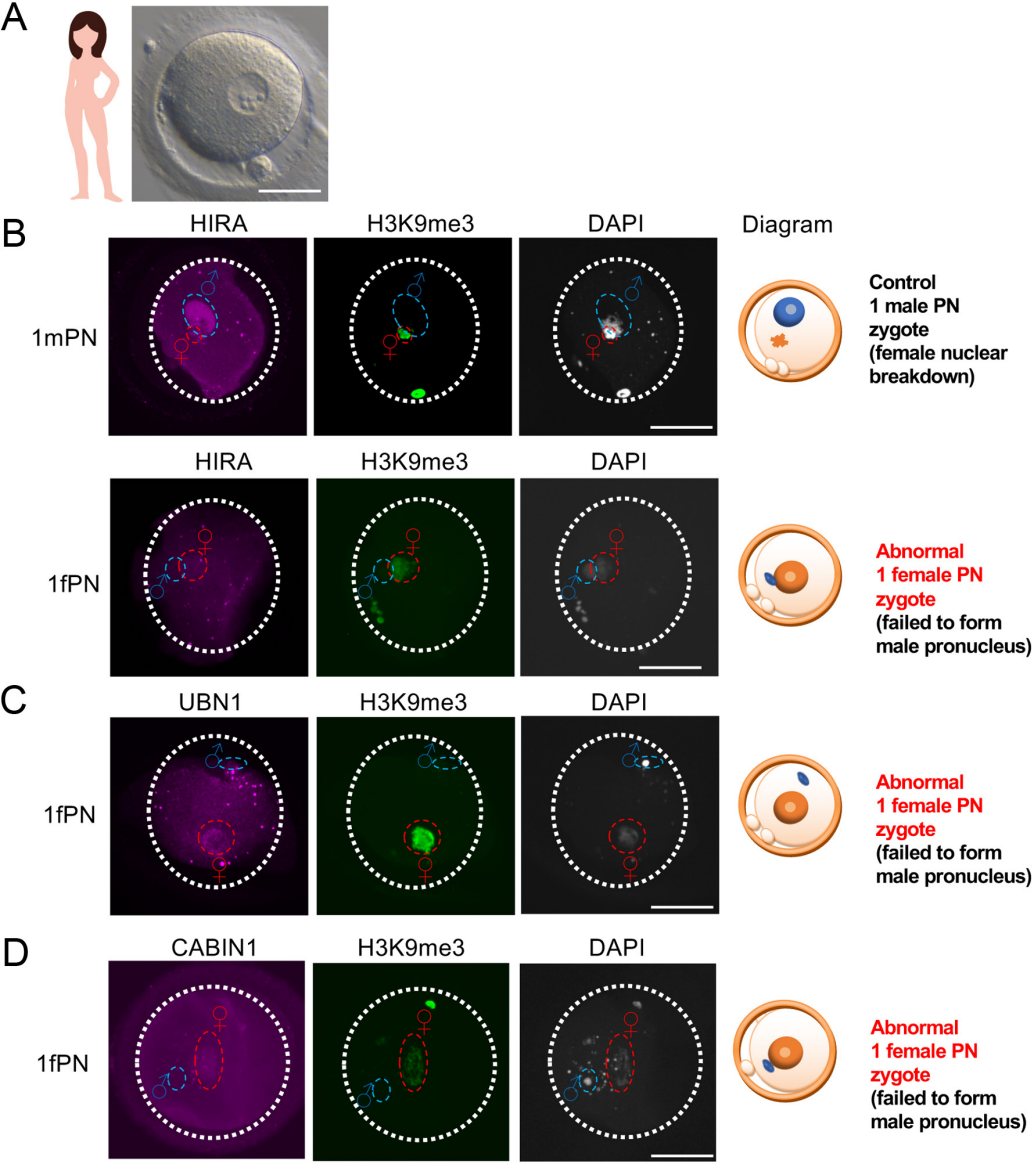
One-pronucleus (1PN) human zygotes revealed deficit of HIRA complex incorporation into the male chromatin. (A) Representative image of a collected human 1PN zygote collected. (B) HIRA failed to associate with the male chromatin of abnormal female 1PN zygote. Immunofluorescence of HIRA and H3K9me3 of human 1PN zygotes. HIRA associates with the male chromatin of a normally fertilised zygote (female chromatin progressed to nuclear breakdown, while male PN remained; upper panel), but failed to associate with the abnormal female 1PN zygote (failure of male pronucleus formation; lower panel). (C) UBN1 failed to associate with the abnormal 1 female PN zygote. Immunofluorescence of UBN1 and H3K9me3 of a human 1PN zygote. UBN1 failed to associate with the male chromatin. (D) CABIN1 failed to associate with an abnormal 1 female PN zygote. Immunofluorescence of CABIN1 and H3K9me3 of a human 1PN zygote. CABIN1 failed to associate with the male chromatin of an abnormal 1 female PN zygote. Histone H3K9me3 labels female chromatin; DNA counterstained by DAPI. Scale bar = 50µm

Human 1PN embryos were donated for research use after examination of zygotes 18 h post insemination (following both IVF and ICSI). During the recruitment period of 2018–2019, we approached a total of 218 couples and 144 couples gave consent. Overall, 23 1PN zygotes were identified from 16 IVF/ICSI cycles and 21 were fixed (1PN /fertilised embryo: 21/101 = 20.1%; Table 1 and Fig. 3A). Subsequent analysis showed that an additional nine embryos originally identified in the clinic as being 1PN were at more advanced stages upon collection. Seven had progressed to nuclear envelope breakdown (NEBD) or the 2-cell stage, and two were parthenogenetically activated.

**Table 1:** Summary of the one-pronucleus zygote collection during recruitment period.

**Recruitment period**	**Patients**, *n*			**1PN zygotes**, *n*	
	Approached	Consented	Showing 1PN zygotes	Collected	Used^†^
2018	70	46	8	9	9
2019	148	98	8	14	12
Total	218	144	16	23	21*

^†^Used for experiments; *Not all zygotes were found to be 1PN on assessment in the laboratory, nine were at other stages, specifically one-male pronucleus zygotes as control x2; wrong stages x5, and parthenogenesis x2.

We performed IF on the fixed samples using H3K9me3, a female chromatin specific marker in human (van de Werken *et al.* 2014), in order to distinguish the origin of the parental chromatin. We identified that there were two 1PN zygotes which were actually morphologically abnormal but cytologically normal diploid zygotes. The parental chromatin had developed asynchronously: the female chromatin had progressed to nuclear envelope breakdown stage, while the male chromatin had remained in the pronuclear stage (Fig. 3B; also observed in Egli *et al.* (2011)). In these one male PN zygotes, we observed that the HIRA immunostaining signal was enriched (Fig. 3B). In contrast, in the abnormal one female PN zygotes (female chromatin labelled by H3K9me3 (van de Werken *et al.* 2014)), the male chromatin had failed to form the male pronucleus, remaining in the decondensation stage, and importantly lacking HIRA staining. This is in agreement with the data collected from *Hira* mutant mice (Fig. 2A).

As in the loss-of-function *Ubn1* fertilised 1PN phenotype in the mouse, we also found that UBN1 was absent in the male chromatin of the 1PN human zygotes (Fig. 3C). We noted that in half of the 1PN zygotes we stained for CABIN1, signal was absent in the male chromatin (Fig. 3D). This was consistent with the abnormal 1PN formation rate in *Cabin1* mutant mice (Fig. 2C). A summary of immunofluorescence results is shown in Table 2.

**Table 2:** Summary of immunofluorescence result of abnormal one-pronucleus zygote.

**Stained antibodies**	**One fPN zygotes stained with antibodies**, *n*	**Positive signal detected in male chromatin**, *n*	**Ratio**
HIRA	4	0	0/4
UBN1	4	0	0/4
CABIN1	4	2*	2/4
Total	12	2	2/12

*One with weak signal.

### Overexpression of Hira in mutant oocytes rescues male pronucleus formation in their zygotes

The results above show that maternal HIRA complex is critical for male pronucleus formation and that loss-of-function leads to abnormal 1PN phenotypes in mice (Fig. 2). In these abnormal phenotypes, HIRA complex fails to incorporate into male chromatin which is likely to contribute to the abnormal 1 female PN human zygote phenotype (Fig. 3). Both *Hira* and *Cabin1* null mouse showed later developmental defects than maternal depletion models (see discussion), thus, maternal deposition of HIRA complex has a more profound role than previously understood during the oocyte-to-embryo transition, and this cannot be compensated by zygotic transcription rescue during the two-cell stage.

**Figure 4:**
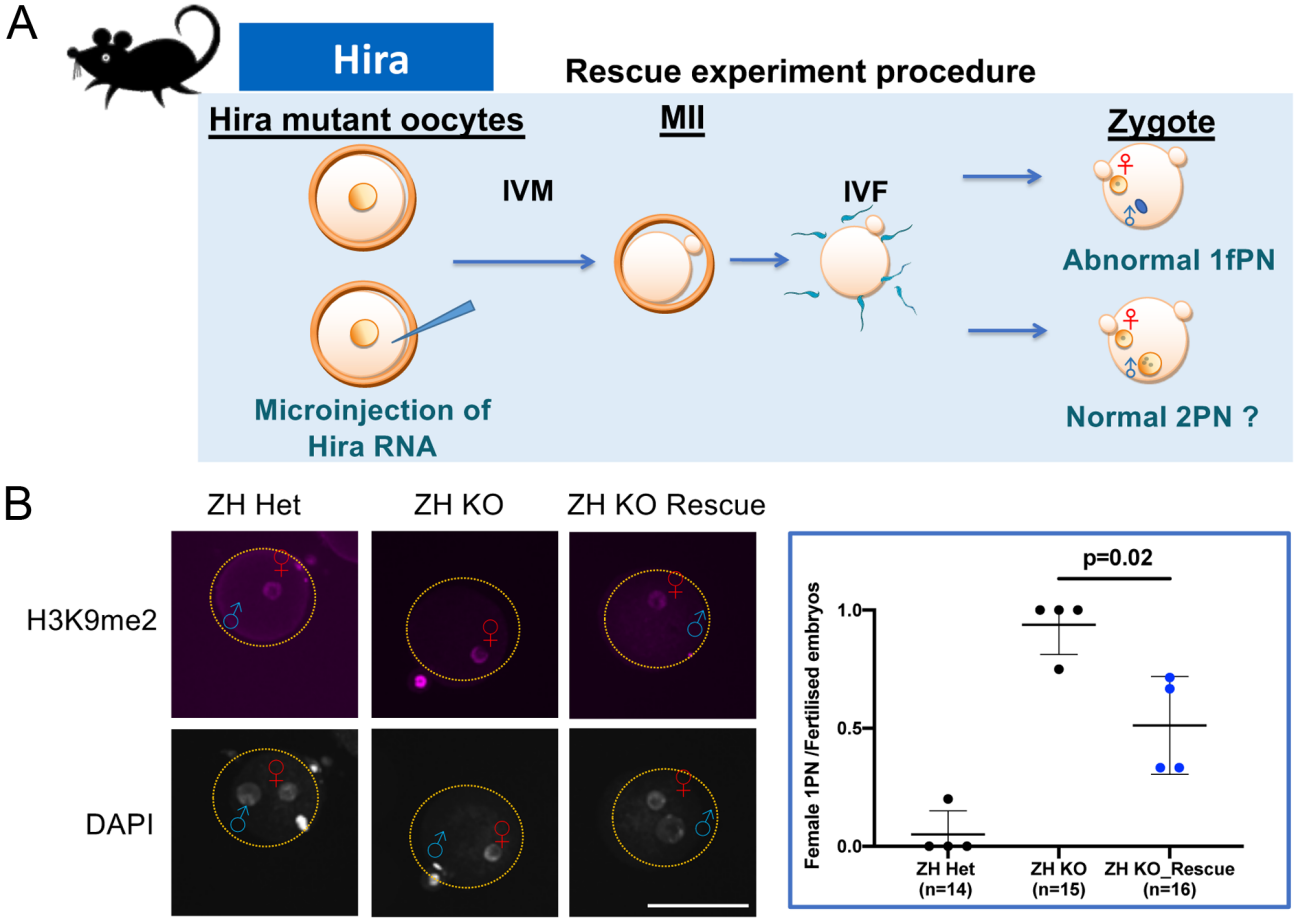
Overexpression of *Hira* rescued the male pronucleus formation in the *Hira* mutants. (A) Experimental procedure for rescuing maternal *Hira* mutants. *Hira* RNA was introduced into the GV oocytes, following maturation and fertilisation *in vitro*. Pronuclear formation was examined by immunofluorescence. (B) Over-expression of *Hira* RNA in the *Hira* mutant oocytes rescued the male pronucleus formation after fertilisation. Immunofluorescence of H3K9me2 showed that zygote with two pronuclei was formed after rescue (left panel). Summary of the rescue experiments (right panel). Scale bar = 80 μm.

We envisaged that abnormal 1PN zygote phenotypes caused by the maternal defect of *Hira* could potentially be rescued by overexpression in oocytes. To test this, we first adapted the same micromanipulation platform as we applied to knockdown of *Ubn1* in oocytes (Fig. 4A). Instead of morpholino oligos (Supplementary Fig. 2B), we injected *in vitro* transcribed RNA into *Hira* mutant (ZH KO) oocytes. The GFP-tagged HIRA protein was stably expressed after culture, and we were able to increase HIRA in the mutant oocytes to a level comparable with that of *Hira* heterozygous (ZH Het) oocytes ((Supplementary Fig. 3); ZH Het from significant difference of HIRA level of Het vs KO *P* = 0.023 to no significance of Het vs KO-injected). On the other hand, the level of H3.3 showed no significant change which indicated that HIRA overexpression does not alter overall H3.3 expression, while still enabling its histone chaperoning ability of loading H3.3 incorporation into chromatin.

We then introduced *Hira* RNA into *Hira* mutant oocytes. *In vitro* maturation was conducted, and mature oocytes were next fertilised *in vitro*. To facilitate the fertilisation process not hampered by zona hardening during prolonged culture and address whether penetrated sperm heads can properly decondense (Lin *et al.* 2014), we removed zona pellucida before *in vitro* fertilisation. We determined formation of the male pronucleus as an indicator of a successful rescue event by IF (Fig. 4B). ZH KO fertilised embryos showed a high proportion of abnormal one female PN zygotes compared to ZH Het zygotes (86.7% vs 7%, *P* = 2.7E-17). However, in ZH KO oocytes with *Hira* RNA injection partially rescued the proportion of abnormal one female PN zygotes from 86.7 to 56% (*P* = 0.02; Fig. 4B). This result clearly supports our assertion that overexpression of HIRA in null oocytes can support zygote formation, thus providing further support for the proof-of-principle approach to rescuing poor quality oocytes (here, null *Hira*) in order to support oocyte-to-embryo transition.

## Discussion

We have demonstrated, that in mice, *Cabin1* and *Ubn1* are maternal factors which are associated with decondensed male chromatin after fertilisation and physically interact with HIRA. We showed that molecules within the HIRA complex, CABIN1 (a mammalian specific H3.3 chaperone (Orsi *et al.* 2013)) and UBN1, acting interdependently, are also critical for male pronucleus formation in mouse. The roles of *H3.3* and *HIRA* have been reported in bovine pre-implantation stages (Zhang *et al.* 2016,^2018^), but relevant study, particularly focusing on maternal roles, has been lacking. Improved understanding will be beneficial for the improvement of domestic animal production. Importantly, our investigation of abnormal human 1PN zygotes showed that HIRA complex was absent in the male chromatin, thus linking this unique phenotype with a potentially coherent mechanism of zygote formation in mice and humans. Using our mouse model, we showed that we could ameliorate the deleterious effects of the loss of *Hira*, thus providing proof of concept of rescue as a potential therapeutic approach.

Results of molecular cytogenetics of human 1PN zygotes have been reported. These results show that the possible underlying causes include synchronous PN formation, fusion of maternal and paternal PN, parthenogenetic activation, or premature breakdown of PN (Azevedo *et al.* 2014). In our limited but carefully phenotyped 1PN samples (*n* = 21) we observed that the majority showed failure of male pronucleus formation (Fig. 3), indicating an abnormal fertilisation event. Other zygotes, initially identified as having 1PN, were subsequently shown to have asynchronous PN formation (*n* = 2; as the IF controls), parthenogenetic activation (*n* = 2), or incorrect staging (progressed to nuclear breakdown and two cells; *n*  = 5) (Table 1). Our results support the statement that, in general, 1PN zygotes should be considered to have undergone abnormal fertilisation and to be less competent of progressing to a normal embryo, (Yao *et al.* 2016, Araki *et al.* 2018, Destouni *et al.* 2018).

Originally, null mutants demonstrated the requirement for *Hira* around the time of gastrulation (Roberts *et al.* 2002) but our previous studies discovered that maternal *Hira* is essential for zygote development (Lin *et al.* 2014, Nashun *et al.* 2015). Similarly, *Cabin1* null mice showed embryo lethality around embryonic day 12.5 due to organogenesis defects (Esau *et al.* 2001). Interestingly, our oocyte-deleted *Cabin1* mutants revealed pre-implantation defects (Smith * et al.*^ 2020^ bioRxiv). Based on the results of our *Hira* maternal mouse models, which revealed a great loss of developmental propensity (Lin *et al.* 2014), we envisaged that the lower developmental potential of human 1PN embryos might be due to defects of embryonic genome activation, arising at the 4–8 cell stages in humans. It would be valuable to measure the transcriptome of abnormal 1PN zygotes to establish a profile of the downstream effectors as a 'good oocyte' signature. Also, it will be valuable to monitor the subsequent pregnancy outcomes from patients who donated the 1PN zygotes and the reoccurrence of 1PN zygotes after new cycles.

Next, we mined the human exon sequence dataset, GenomeAD Browser (https://gnomad.broadinstitute.org), to examine whether there is a genetic linkage relating to the loss-of-function of HIRA complex. We noted that the loss-of-function observed/expected upper bound fraction (LOEUF) parameters of *HIRA* (0.02–0.14) and *UBN1* (0.05–0.21) are very low (*CABIN1* (0.36–0.59)), indicating low tolerance to inactivation of the genes. In other words, both *HIRA* and *UBN1* likely tend to result in loss-of-function mutations. The interdependence of HIRA complex components shown in Supplementary Fig. 2D and E, combining the results from Nashun * et al.* (Nashun *et al.* 2015) and the previous data in human cells (Rai *et al.* 2011) indicate that the normal function of all three components of HIRA complex is required for its adequate function. Similarly, the abnormal human 1PN zygotes we collected were possibly inherited from loss-of-function *HIRA* complex oocytes. This means that abnormal 1PN zygotes might harbour divergent transcriptomic and chromatin profiles. Therefore, it will be of interest to collect human 1PN zygotes and perform RNA-sequencing or chromatin-associated assays (ATAC-seq (Wu *et al.* 2016) and Hi-C (Flyamer *et al.* 2017) or scNMT-seq (Clark *et al.* 2018)) to gain insight into human paternal genome reprogramming events.

Overexpression of *Hira* mRNA in mutant murine oocytes partially rescued the abnormal 1PN phenotype by significantly restoring mutant oocyte fertilisation to form normal two PN zygotes (Fig. 4). This not only demonstrated that maternally stored HIRA is critical for male pronucleus formation but also provided a proof-of-principle experiment as the foundation of an approach for rescuing defects caused by impaired maternal factors. A rescue attempt of *Hira* 1PN zygote phenotype has been showed by co-injection of *Hira* siRNA with mRNA during oogenesis (Inoue & Zhang 2014). Our new rescue result reinforces the applicability of this approach. We introduced *Hira* mRNA into fully grown *Hira* mutant GV oocytes, the end stage of oogenesis, and partially restored the abnormal 1PN zygote phenotype to normal phenotype. Since Zp3-Cre driven recombination happens during oogenesis, both maternal *Hira* mRNA and protein have been depleted. The consequent feasibility of rescuing mutant oocytes at an advanced oocyte stage directly demonstrates the expansion of the window of opportunity to rescue defective maternal effect genes. Also, fully grown GV oocytes are the ideal stage for oocyte manipulation compared to growing oocytes.

Besides the likelihood of the HIRA-mediated H3.3 incorporation mechanism being directly involved in paternal chromatin assembly, Gou *et al.* (2020) reported that *Srpk1*, a splicing kinase, not only acts as a key factor in phosphorylating protamine to initiate paternal genome programming but it also facilitates protamine interaction with HIRA for H3.3 deposition. It will be worth investigating whether CABIN1 or UBN1 is associated with SRPK1, and whether SRPK1 is the upstream regulator to initiate the nucleosome assembly process. Another possible avenue would be to examine the overall transcriptional impact of HIRA complex in the mutant oocytes, in order to investigate whether there is an effect on deposition of maternal factors during oogenesis (e.g. *Srpk1* or others). On the other hand, careful dissection of the downstream consequences of absence of HIRA complex, such as profiling the global chromatin landscape by genome-wide approaches, examining the formation of nuclear pore envelope assembly after failure of nucleosome assembly (Inoue & Zhang 2014) or further determining the transcriptional impact, require further endeavour. These future studies will not only lead towards a better understanding of the mechanism of action but also advance future clinical diagnosis and identification of potential biomarkers of the 1PN phenotype in the human.

In conclusion, by generating new loss-of-function mouse models and analysing human 1PN zygotes, we report new molecular insights into human 1PN zygote formation.

## Supplementary Material

Supplementary Figure 1. Proximity ligation assay shows the positive interaction of HIRA complex molecules in the mouse oocytes. (A)UBN1 and HIRA reveal positive interaction in the GV nuclei by PLA assay. Control groups are UBN1 alone or UBN1 with hCG. Right panel; quantification of PLA foci. (B) CABIN1 and HIRA reveal positive interaction in the GV nuclei by PLA assay. Control groups are CABIN1 alone or CABIN1 with hCG. Right panel; quantification of PLA foci.

Supplementary Figure 2. Interdependence of HIRA complex molecules in the mouse oocytes.

Supplementary Figure 3. Validation of HIRA overexpression in the Hira mutant oocytes. Immunofluorescence of HIRA and H3.3 of the Hira mutant (ZH KO) oocytes after RNA injected (left panel). Quantification of immunofluorescence result showed that the level of HIRA in the Hira mutant oocytes was comparable to heterozygous controls (ZH Het); right panel. Scale bar=80µm.

## Declaration of interest

The authors declare that there is no conflict of interest that could be perceived as prejudicing the impartiality of the research reported.

## Funding

This work was supported by MRC Centre Grant MR/N022556/1, the Wellcome Trust http://dx.doi.org/10.13039/100010269 -University of Edinburgh Institutional Strategic Support Fund, Barbour Watson Trust, and grants from Deanery of Clinical Sciences, College of Medicine & Veterinary Medicine of University of Edinburgh to C-J L. C-J L is a Royal Society http://dx.doi.org/10.13039/501100000288 of Edinburgh Personal Research Fellow funded by the Scottish Government http://dx.doi.org/10.13039/100012095 .

## Author contribution statement

R S involved in mouse colony management and performed oocyte experiments. S J P and A K performed human IVF treatment under the overall management of K J T. R A A is a HFEA licence holder. C-J L, R S, and R A A interpreted the data. C-J L conceived the project and designed the experiments, performed all embryo micromanipulation and human IF work. R S, R A A, and C-J L wrote the manuscript with input from all authors.
