# Supplementary material for: HIRA contributes to zygote formation in mice and is implicated in human 1PN zygote phenotype
Source: Reproduction. 2021 Apr 8;161(6):697–707. doi: 10.1530/REP-20-0636 (PMC8188263; doi:10.1530/REP-20-0636)
Supplement: Supplementary Figure 1. Proximity ligation assay shows the positive interaction of HIRA complex molecules in the mouse oocytes. (A)UBN1 and HIRA reveal positive interaction in the GV nuclei by PLA assay. Control groups are UBN1 alone or UBN1 with hCG. Right panel; quantification of PLA foci. (B) CAB [file supplementary_figure_1.pdf]

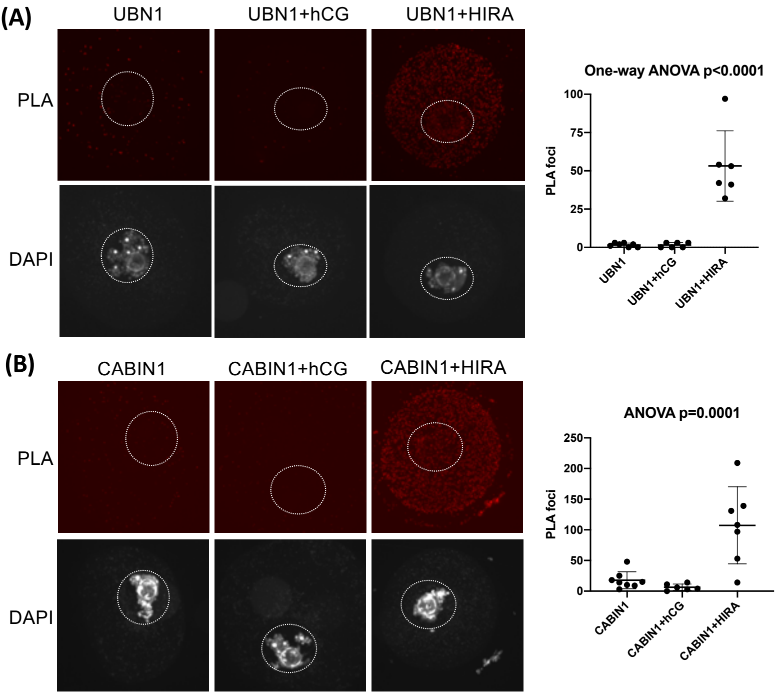

Supplementary Figure 1. Proximity Ligation Assay shows the positive interaction of HIRA complex molecules in the mouse oocytes.
