# Supplementary figures and images for: HIRA contributes to zygote formation in mice and is implicated in human 1PN zygote phenotype
Source: Reproduction. 2021 Apr 8;161(6):697–707. doi: 10.1530/REP-20-0636 (PMC8188263; doi:10.1530/REP-20-0636)

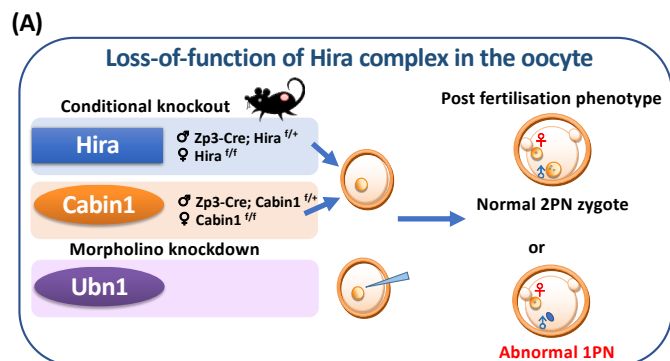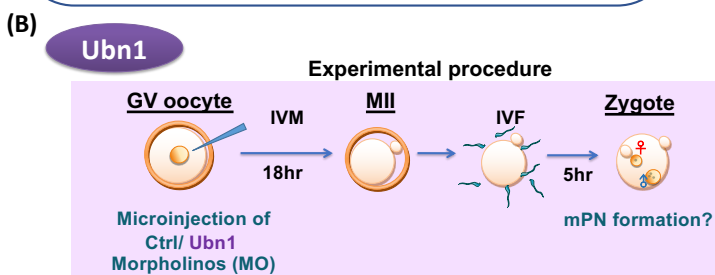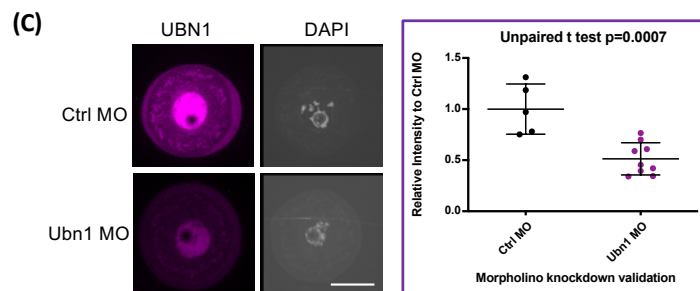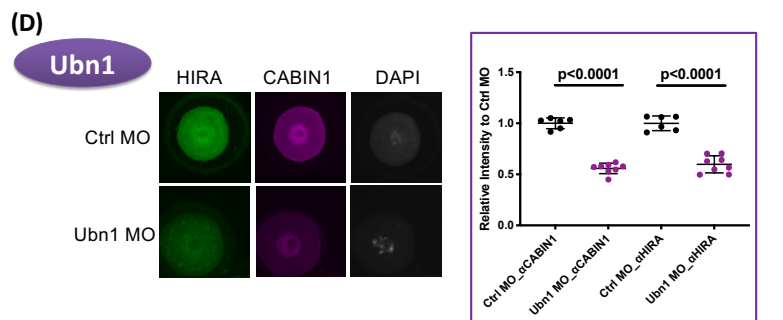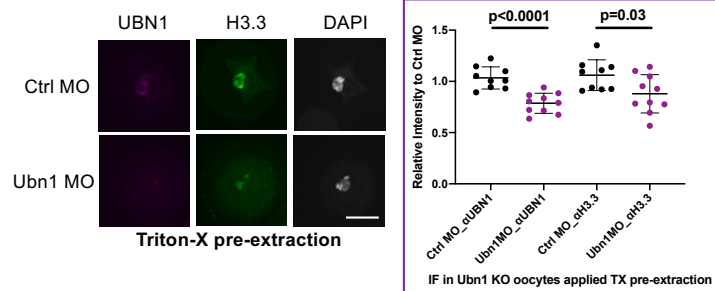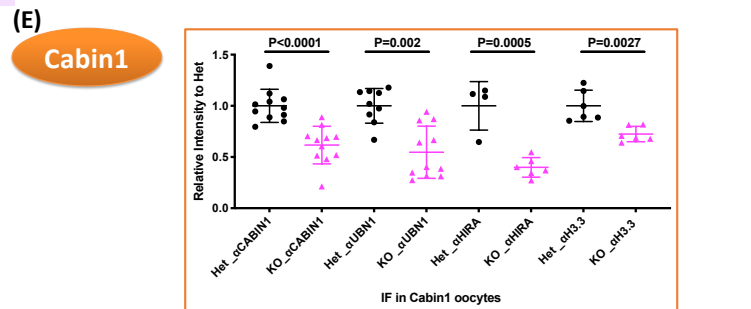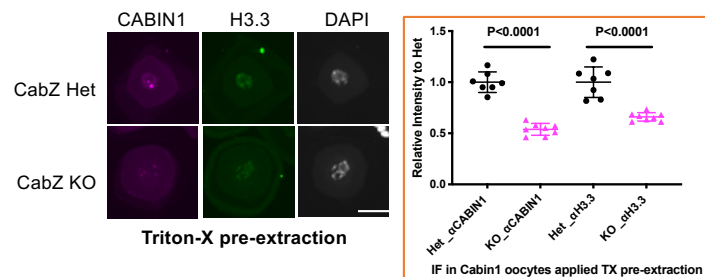

Supplementary Figure 2. Interdependence of HIRA complex molecules in the oocytes.

Supplement: Supplementary Figure 2. Interdependence of HIRA complex molecules in the mouse oocytes. [file supplementary_figure_2.pdf]

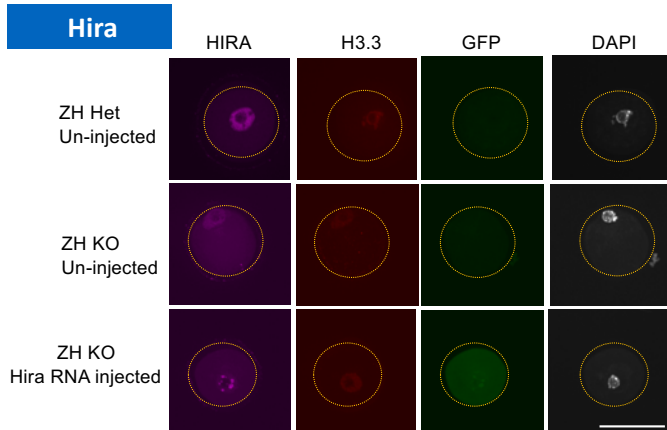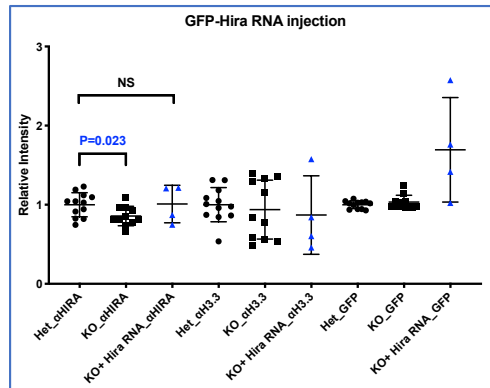

Supplementary Figure 3. Validation of HIRA overexpression in the Hira mutant oocytes

Supplement: Supplementary Figure 3. Validation of HIRA overexpression in the Hira mutant oocytes. Immunofluorescence of HIRA and H3.3 of the Hira mutant (ZH KO) oocytes after RNA injected (left panel). Quantification of immunofluorescence result showed that the level of HIRA in the Hira mutant oocytes was compa [file supplementary_figure_3.pdf]
